# A Feasible Strategy for Fabricating Surface Porous Network in Fe-Si Ribbons

**DOI:** 10.3390/ma11050701

**Published:** 2018-04-29

**Authors:** Shuai Wang, Biao Chen, Yongfeng Liang, Feng Ye, Junpin Lin

**Affiliations:** State Key Laboratory for Advanced Metals and Materials, University of Science and Technology Beijing, Beijing 100083, China; wangshuaizrr@126.com (S.W.); chenbiaoustb@126.com (B.C.); yefeng@skl.ustb.edu.cn (F.Y.); linjunpin@ustb.edu.cn (J.L.)

**Keywords:** surface porous network, selective electrochemical dissolution, Fe-Si alloy, cellular structure

## Abstract

Porous materials have always attracted extensive attention owing to their low density, tunable porosity and high surface area. Generally, porosity is introduced in amorphous materials through dealloying or electrochemical dealloying processes. In this work, an iron-based surface porous network was successfully fabricated utilizing selective electrochemical dissolution of Fe-Si alloy ribbons based on the cellular structure prepared by melt-spinning technique. After 30 s, the surface of the ribbon gradually becomes flat and grains can be observed in the first stage of electrochemistry; after an extra 10 s, the pores spread throughout the surface of the ribbon in the second stage. The average size of pores is about 310 nm and the average size of the ligament is 150 nm. The associated dissolution mechanism has been proposed based on the inhomogeneous composition of the center and edge of the cell. The entire process of electrochemical dissolution has been divided into two stages and the entire duration of synthesis does not exceed one minute. This method is extremely feasible and provides a promising strategy for preparing surface porous materials for selective electrochemical dissolution of cellular structure.

## 1. Introduction

Nowadays, porous materials attract significant attention and research interest owing to their high surface area, low density and tunable porosity, which finds several applications in the fields of catalysis, energy storage, sensing, etc. [1,2,3]. As such, the application prospects of the porous materials are extensive. Many studies are entirely devoted to the preparation of various kinds of porous materials, such as platinum (Pt), gold (Au), silver (Ag) and copper (Cu) [4,5,6,7,8]. Furthermore, Fe-based porous materials have also attracted increased attention due to their considerable economic benefits and cost effective performance [9,10,11] compared with precious metal based porous materials. The above mentioned porous materials have been prepared based on the dealloying process of amorphous alloys or bi-phase alloys which are used as precursor samples. Generally, the dealloying process is time-consuming.

In the present work, Fe-Si alloy ribbons with cell array structures have been used as a precursor to successfully fabricate the iron-based porous network by process of selective electrochemical dissolution. Studies about preparing porous materials based on the inhomogeneous composition of the center and edge of the cell have rarely been reported. The process is highly efficient and time-saving. The entire duration of synthesis does not exceed one minute. Fe-Si alloy ribbons containing a single phase were prepared by a melt-spinning technique. The electrochemical dissolution processing of Fe-Si alloy ribbons emerges as an efficient and controllable synthesis procedure.

## 2. Materials and Methods

The Fe-6.5 wt % Si ribbons have been prepared by melt-spinning in accordance with our previously reported study [12]. A two-electrode DC power system was employed to conduct the electrochemical experiments and a platinum foil was used as the counter electrode. A perchloric acid alcohol solution with 0.6 M concentration was used as the electrolyte. The distance between the electrodes was maintained as 30 mm and a voltage of 30 V was applied (in this case, the current is approximately 996 mA). The electrochemical dissolution of the Fe-Si ribbons was performed in perchloric acid alcohol electrolyte having a concentration of 0.6 M at 298 K for different time intervals ranging from 2 to 40 s. After complete dissolution, the samples were rinsed several times with distilled water and dehydrated alcohol.

A Zeiss Supra™ 55 field emission scanning electron microscope (Carl Zeiss AG, Oberkochen, Germany) was used to observe the morphology on the surface of the ribbons. The microstructure and composition of the samples were analyzed using transmission electron microscopy (FEI Tecnai G2 F30 TEM, FEI Corporation, Hillsboro, OR, USA) equipped with an energy dispersive X-ray spectroscopy unit (FEI Corporation). The phase of Fe-Si ribbons was identified through the X-ray diffraction (XRD, EMPYREAN, Panalytical Co., Ltd., Almelo, The Netherlands) with Cu-Kα radiation.

## 3. Results and Discussion

**Figure 1:**
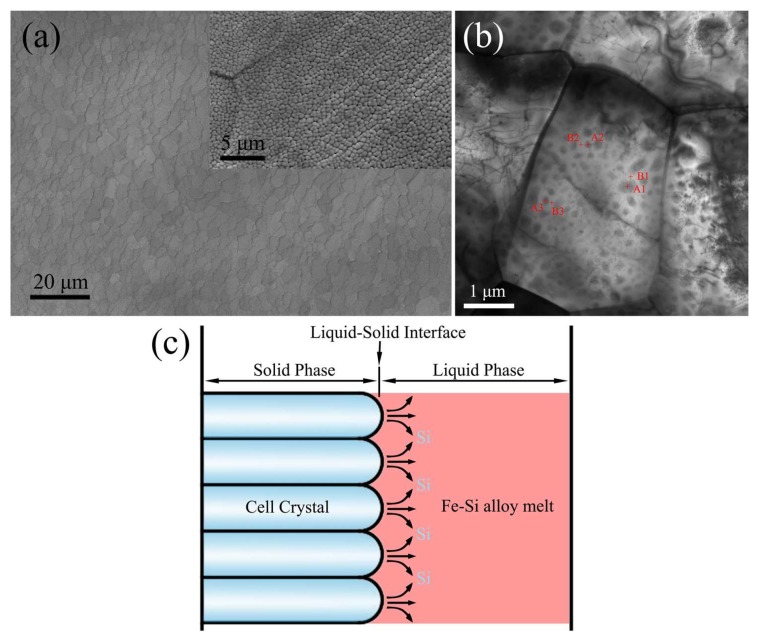
(**a**) SEM morphologies of the free surface of Fe-6.5 wt. % Si ribbons; (**b**) light-field image of Fe-6.5 wt. % Si ribbons; (**c**) schematic diagram depicting growth of cell.

In the paper, the ribbons with 20 mm in width and 35 μm in thickness were prepared by ejecting the molten alloy on a copper wheel with 20 cm. The rotation speed of copper wheel was 20 m/s. In Figure 1a the free surface morphology of the Fe-Si alloy ribbons prepared by melt-spinning has been represented and the composition of the ribbons measured by chemical analysis was Fe: 93.54 wt %, Si: 6.46 wt %. The cellular array structure can be seen on the free surface as indicated in the inset of Figure 1a. The average size of the cell is obtained as approximately 400 nm. Figure 1b depicts the bright-field image of TEM where the contrast due to component differences between the center and edge of the cell crystal is clearly visible. The energy spectrum corresponding to TEM has been investigated to analyze the components distributed between the cell’s center and edge. The as-obtained results are listed in Table 1. A1, A2, and A3 represent the components of the cell’s center, while B1, B2, and B3 represent the components of the cell’s inter-granular zones. The content of silicon at the cell’s center is generally lower than that in between them. From Figure 1c, it is evident that this is due to solute redistribution during solidification. The front of the cell in the solid-liquid interface will discharge solute Si towards the periphery, resulting in the differences in the composition of the center and the edge of the cell. Cellular crystal formation and cell size are closely related to the conditions of solidification [13,14] and that is, the cell size can be regulated by changing the cooling conditions.

**Figure 2:**
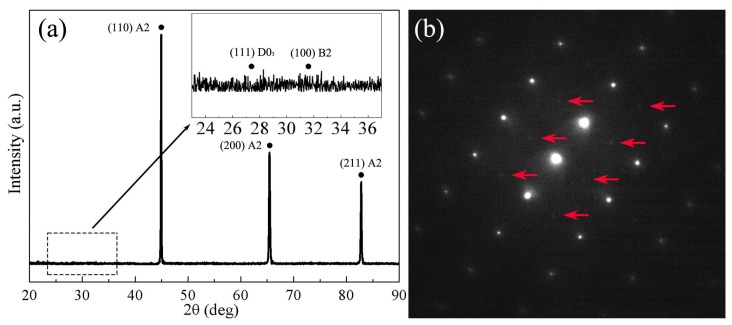
(**a**) XRD patterns of Fe-6.5 wt % Si ribbons; (**b**) TEM diffraction spots in the 011 zone.

Figure 2a shows XRD patterns of Fe-Si alloy ribbons fabricated by melt spinning process. XRD pattern of Fe-Si alloy ribbons consisting of the fundamental diffraction peaks in (220), (400) and (422), where A2, B2 and D0_3_ have the peaks at the same positions without any glassy structural characteristic. No obvious ordered phases (B2 and D0_3_ structure) super-lattice diffraction peaks can be found within the zoomed-in X-ray diffraction pattern as shown in Figure 2a. The TEM diffraction spot indicates the presence of B2 (the red arrows indicate locations), but the signal is quite weak. The XRD and TEM results indicate that the generation and transformation of the ordered phases can be effectively suppressed, which is in agreement with results previously reported in the literature [15].

**Figure 3:**
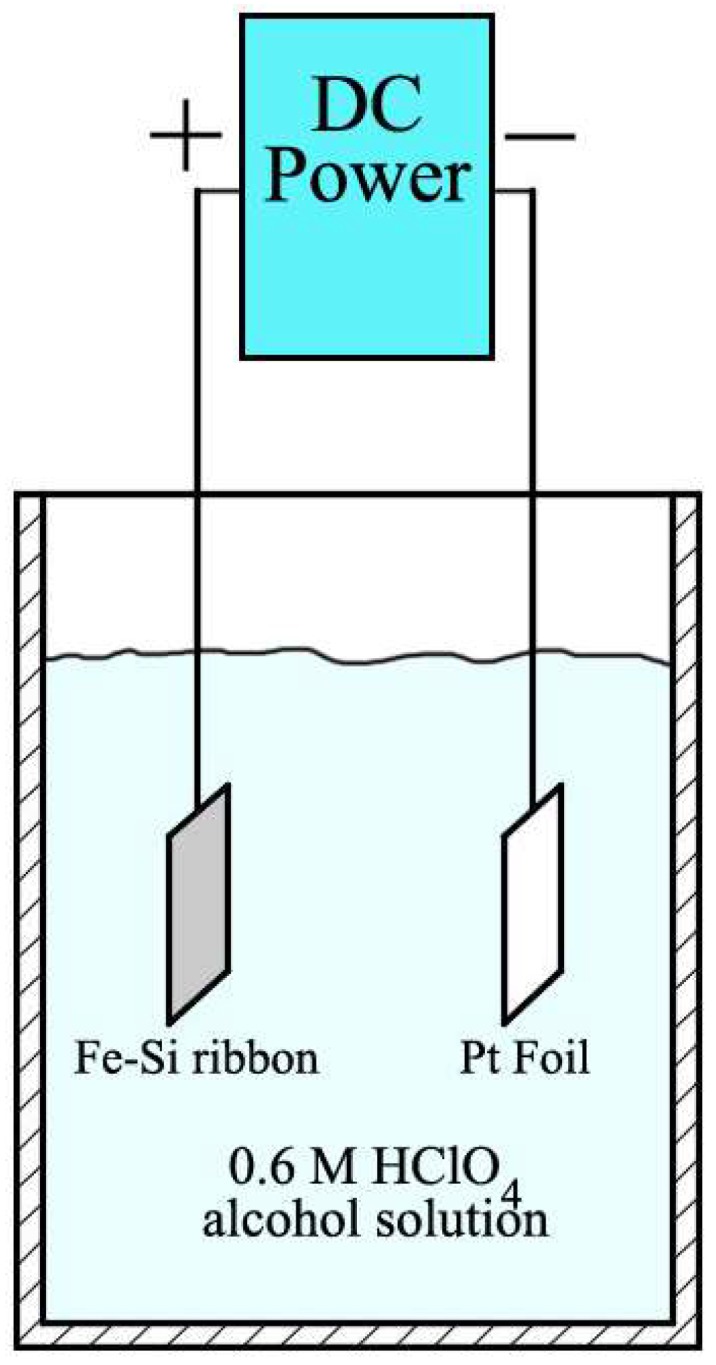
Schematic diagram depicting the process of electrochemical dissolution. The reaction vessel of electrochemical dissolution is a 500-mL beaker containing 400 mL 0.6 M HClO_4_ alcohol solution.

The Fe-Si alloy ribbons possessing a cell array structure and without containing any glassy structural characteristics were used as precursors to prepare the iron-based surface porous network through the process of anodic dissolution in 0.6 M HClO_4_ alcohol solution (Figure 3). For this purpose, the Fe-Si ribbon is connected to the positive pole of the DC power, and the Pt foil and the cathode are connected to form the negative side. The distance between the positive and negative electrodes is maintained at 3 cm. Subsequently, the anodic dissolution of Fe-Si ribbons of different durations are used to observe the synthesis process. The entire electrochemical dissolution process can be divided into two stages.

**Figure 4:**
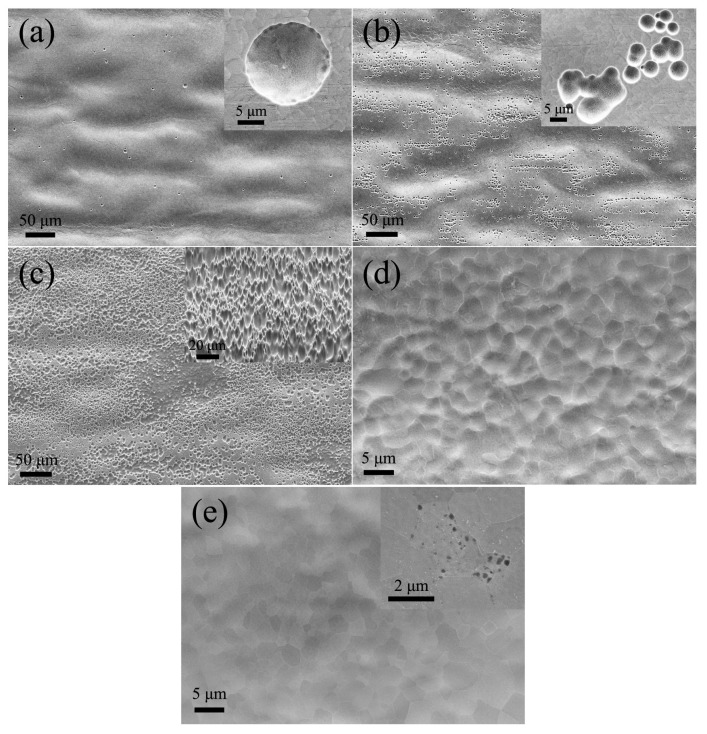
Surface topography after dissolution process corresponding to a duration of (**a**) 2 s; (**b**) 5 s; (**c**) 10 s; (**d**) 20 s and (**e**) 30 s. (**a**–**c**,**e**) insets are magnified pictures of the corresponding picture.

Figure 4a shows that the sporadic dissolution results in formation of holes on the surface of the ribbon during the 2 s electrochemical process. Figure 4a inset depicts the magnified topography. The sizes of the micropores range from 3 to 10 microns. Most of the pores appear on the convex surface of the ribbon, which is closer to the Pt electrode. This phenomenon is clearly shown in Figure 4b. Due to the increase in the number of holes generated by increasing the duration of electrochemical processing, the edges of the holes grow towards forming a contact with each other and start to form a connected piece together. Ten seconds after subjecting the sample to electrochemical treatment, the holes are distributed all around the surface of the ribbon, irrespective of convex or concave areas on the surface of the ribbon. This is shown in Figure 4c. In some areas there are no separate micro-holes shown, where the holes have been inter-connected. The resulting surface of the ribbon becomes uneven due to the appearance of increasing number of micropores shown in the inset of Figure 4c, which was obtained by tilting the sample surface by 30° (that is, the angle is 60° between electron gun (or electron beam) and sample surface). Figure 4d shows that after a longer period of electrochemical treatment (20 s), the holes on the surface of the ribbon have been inter-connected. At this time there are still some fluctuations on the surface of the ribbon (as seen in Figure 4d). After an additional processing of 10 s, the surface of the ribbon becomes considerably smooth and its associated grain structure can be observed in Figure 4d. The holes with dimensions ranging between 80 and 300 nm begin to appear in some areas, as shown in the Figure 4e inset.

We define the above process as the first stage. The macroscopic morphology of the ribbon predominates during the first stage of electrochemical dissolution and the height difference of the macroscopic morphology gradually decreases with the progress of electrochemical dissolution process. Finally, this dissolution process gradually becomes a process controlled by the difference in composition along the radial direction of the cell crystal. This marks the beginning of the second stage of dissolution. The next process is the second stage of electrochemical dissolution.

**Figure 5:**
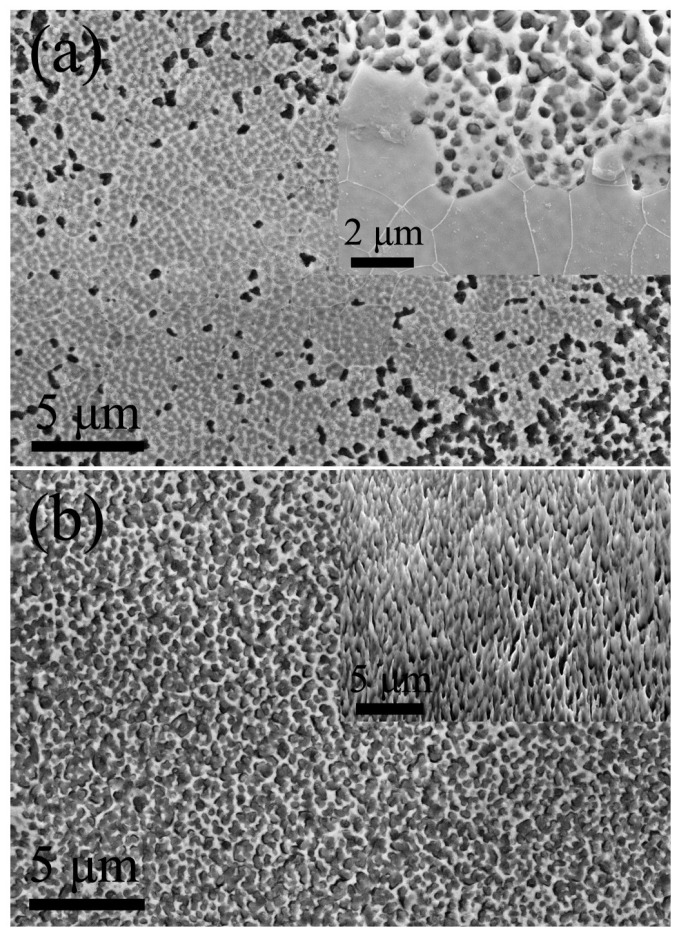
(**a**) Initial stage and (**b**) the accomplishment of the formation of porous surface network. (a,b) insets are magnified pictures of the corresponding picture.

After 35 s of electrochemical dissolution, as shown in Figure 5a, the surface state which depicts a transition from the metallurgical surface (Figure 4e) to the porous surface (Figure 5b). From the Figure 5a inset, the surface gradually erodes and the pores are with an average size of 280 nm and a ligament having an average dimension of 260 nm emerges. The pore/ligament ratio obtained is 1.1. This was mainly due to the presence of the different electrode potentials caused by the inhomogeneity in the components within and outside the cell.

Owing to the prolonged dissolution time, the pores tend to spread throughout the surface of the Fe-Si ribbon after 40 s, as shown in Figure 5b. The average size (310 nm) of the pores is notably increased compared to the sample depicted in Figure 5a, and the average size (150 nm) of the ligament is significantly reduced while the pore/ligament ratio increases from 1.1 to 2.1. The appearance of pores significantly increases the surface area of the ribbon, which is more explicitly shown in Figure 5b inset.

In conclusion, during the whole electrochemical dissolution process, the sporadic pores begin to appear on the surface of Fe-Si ribbon after 30 s and the size of pores ranges between 80 and 300 nm. After 35 s, many pores emerge on the surface but there are still non-porous areas. The average pore size is 280 nm and the average ligament size is 260 nm. After 40 s, the surface of the ribbon is almost full of pores. The average size of the pores is 310 nm and the average size of the ligament is 150 nm.

These two stages are completed in less than one minute without the addition of any liquid nitrogen to control temperature during the entire process. This method is efficient for preparing superficially porous structures. This synthesis protocol of iron-based porous surface is highly efficient, time-saving and cost-effective. The electrochemical dissolution of Fe-Si alloy ribbons based on their cellular structure appears to be a promising method for fabricating iron-based surface porous networks.

In addition, metal oxides could also be plated into the porous materials. These composites with excellent performance are promising for application in electrode/collector materials [16,17]. The transition metal oxides [18,19,20] are considered to be promising candidates for anode materials owing to their high electrochemical capacities. Many studies on iron oxides have also been performed with the aim of improving the battery performance of cathode materials [21,22] and removing anionic and aromatic dyes from wastewater [23].

Therefore, in the future, the materials used in this work may be used as precursors for preparing iron-based oxides. Ferromagnetic materials can be used as catalysts (or as a carrier for the catalyst) for wastewater purification owing to its advantage of recyclability for avoiding secondary pollution. Hence, the iron-based surface porous network may be promising as catalysts, carriers for the catalyst or cathode.

Moreover, this method may also be a promising route for preparing iron-based surface porous network through efficient selective electrochemical dissolution of Fe-Si alloy ribbons based on the cellular structure prepared by the process of melt-spinning. In the future, the size of the cells may be tuned by changing the composition of the alloy and the cooling conditions of melt-spinning to modify the surface porous network.

## 4. Conclusions

In this work, an electrochemical etching method was used to prepare the iron-based porous surface. The Fe-Si alloy ribbon was used as the precursor and the cell array was prepared by the melt-spinning technique. The entire electrochemical dissolution process can be divided into two stages, which are controlled by the difference between the surface morphology of the ribbon and the composition of the cell, respectively. After 30 s, the surface of the ribbon gradually becomes flat and grains can be observed in the first step of electrochemistry; after an extra 10 s, the pores spread throughout the surface of the ribbon in the second step. The average size of pores is about 310 nm and the average size of the ligament is 150 nm.

The ribbons with the cell array are ideal and feasible precursors to prepare porous surfaces in an efficient manner. In future, we may adjust the size of the cell by changing the composition of the precursor and controlling the cooling conditions and thereby obtaining different sized porous surfaces.

## Figures and Tables

**Table 1:** Components of cell’s center and edge.

Site	Weight (%)	
	Fe	Si
A1	93.27 ± 1.15	6.72 ± 0.31
B1	93.17 ± 1.28	6.82 ± 0.36
A2	94.71 ± 2.02	5.28 ± 0.44
B2	93.87 ± 1.38	6.12 ± 0.31
A3	94.32 ± 1.22	5.67 ± 0.31
B3	93.81 ± 1.25	6.18 ± 0.34
